# Tularemia and tularemia vaccination induce similar, yet partially distinct, multifunctional T-cell responses

**DOI:** 10.3389/fimmu.2026.1812669

**Published:** 2026-05-19

**Authors:** Helena Lindgren, Kjell Eneslätt, Anders Sjöstedt

**Affiliations:** Department of Clinical Microbiology, Umeå University, Umeå, Sweden

**Keywords:** immune memory, T-cell responses, T-cell subsets, tularemia, tularemia vaccination

## Abstract

Vaccination with the live vaccine strain (LVS) or natural infection with *Francisella tularensis* confers long-lasting protection against re-infection, and it is almost exclusively dependent on cell-mediated immunity. The extent, if any, to which vaccine-induced and infection-induced immunity differ in quality and persistence remains unclear. In this study, we compared human immune responses resulting from LVS vaccination and tularemia infection. Peripheral blood mononuclear cells from vaccinees and convalescent patients were recall-stimulated and analyzed for proliferation, characterization of cytokine-producing memory T cells, and cytokine secretion. Both vaccination and infection elicited robust antigen-specific T-cell activation, accompanied by coordinated cytokine release. Intracellular staining identified multifunctional CD4 and CD8 T cells after both vaccination and infection. Characterization of memory subsets revealed that vaccination primarily induced transitional and effector memory populations, while natural infection generated broader and more persistent responses, including activation of terminally differentiated TEMRA (Terminal Effector Memory T cells re-expressing CD45RA) cells and higher intracellular expression of MIP-1β by CD4 and CD8 cells. These findings demonstrate that both LVS vaccination and natural infection induce efficacious immune responses to *F. tularensis*, as evidenced by the presence of long-lasting, multifunctional T cells, but with partially distinct memory profiles. The broader and more durable responses after natural infection highlight areas of relevance to understand the efficacy of existing and future tularemia vaccines.

## Introduction

*Francisella tularensis*, a Gram-negative bacterium, causes the zoonotic disease tularemia. Its virulence critically depends on the ability to invade and proliferate within professional phagocytes (1, 2). Following phagocytic uptake, *F. tularensis* escapes from the phagosome into the cytosol, where it replicates extensively while evading innate microbicidal mechanisms (1). The bacterium’s capacity to persist intracellularly and suppress host innate immune signaling, concomitantly with a very low infectious dose, form the basis for its exceptional virulence (3, 4). In addition, its intracellular nature renders it inaccessible to humoral immunity, and therefore, protective immune responses are dependent on cell-mediated immunity (CMI) (5).

Tularemia is a rarely reported disease in most countries worldwide; however, it exists in many countries of the Northern Hemisphere and local outbreaks may occur (2). The disease is endemic in some countries, most notably in Sweden, Finland, and Turkey; however, even in these countries, the likelihood of recurrent epidemics in the same geographical area is very low or non-existent (6). This unusual epidemiology renders tularemia a useful model for studies of the longevity of cell-mediated immune responses, since re-exposure is highly unlikely to be a reason for sustained immune responses.

The characteristics of *F. tularensis*-specific immune responses have been extensively investigated for decades in various animal models, unequivocally demonstrating that CMI is essential for protection against this highly virulent intracellular pathogen (5, 7). Activated T cells orchestrate protective immunity primarily through the production of interferon-γ (IFN-γ) and tumor necrosis factor (TNF), which stimulate infected macrophages to restrict intracellular bacterial replication and mediate bacterial killing (8). Studies of human immunity have identified immunospecific T cells persisting for decades after tularemia or tularemia vaccination without evidence of waning (9–11). These cells predominantly exhibit characteristics of effector memory and central memory T cells, reflecting their complementary capacities for rapid effector responses (12). Protective immunity to *F. tularensis* is postulated to depend on multifunctional CD4 and CD8 T cells capable of producing IFN-γ and TNF, thereby enhancing macrophage microbicidal activity and limiting bacterial replication (5).

Findings in a previous study of vaccinees and convalescent patients suggested that their immune responses are highly similar and may be functionally indistinguishable (13). The present study revisits the question of whether subtle differences exist between the immune responses of vaccinees and convalescent patients with tularemia. This issue is of particular importance for vaccine development, as reinfection among previously infected, otherwise healthy individuals is exceedingly rare, demonstrating that natural infection confers protective immunity for many decades against *F. tularensis* (10, 11, 14). Consequently, an effective new vaccine should aim to induce multifunctional memory T cells of comparable quality and functionality as of those generated by natural infection.

The *F. tularensis* live vaccine strain (LVS) has for many decades been widely used in various animal and *in vitro* models as a substitute for virulent *F. tularensis* strains to study pathogenesis and host–pathogen interactions in tularemia, as well as to study mechanisms of vaccine-mediated immune responses (5, 15–17). It has been used under investigational protocols, particularly for laboratory workers and military personnel at occupational risk of tularemia but has never been licensed for general use (18).

Herein, we addressed whether there are qualitative or quantitative differences between the LVS-induced and natural infection-induced immune responses. We observed that both LVS vaccination and natural infection induce strong, multifunctional T-cell immunity to *F. tularensis*, but with partially distinct memory profiles.

## Materials and methods

### Vaccination

All participants were immunized on the same day with the LVS of *F. tularensis* (strain NDBR 101, lot no. 11; National Drug Company, Philadelphia, PA). The lyophilized vaccine was reconstituted in 2.0 mL of sterile water, resulting in a concentration of 2.4 × 10^9^ CFU/mL. Approximately 20 µL was applied to the skin of the upper arm by scarification. Ethical approval was obtained from the Swedish Ethical Review Authority (2019-01567, 2020-01860, and 2023-05165).

### Convalescent patients with tularemia

Samples were obtained from 17 convalescent patients with tularemia. Six of the individuals were included in the 1-month group with reported onset 26–32 days before sampling. Eleven individuals were included in the 12-month group with reported onset 355–387 days before sampling. Fourteen of the patients reported symptoms characteristic of ulceroglandular tularemia, whereas the route of infection was unknown in the remaining three patients.

### Isolation and cryopreservation of peripheral blood mononuclear cells

Peripheral blood (approximately 100 mL per donor) was collected from individuals diagnosed with tularemia either 1 month (*n* = 6; 23–68 years old, mean age 53.8 ± 16.6) or 12 months (*n* = 11; 47–78 years old, mean age 61.4 ± 12.2) prior to sampling, as well as from 11 individuals before vaccination (samples denoted as naïve) and 1, 2, 4, and 12 weeks post-vaccination (26–60 years old, mean age 37.8 ± 9.1). Additionally, peripheral blood was obtained from vaccinated donors with previously verified strong recall responses to *F. tularensis*, hereafter designated as high responders (*n* = 7, 44–66 years old, mean age 57 ± 8.8). The latter group had been vaccinated 10–43 years prior to sampling.

Blood samples were collected in CPT tubes (BD Biosciences, NJ, USA), and peripheral blood mononuclear cells (PBMCs) were isolated according to the manufacturer’s instructions. The cells were resuspended in human serum (Innovative Research, MI, USA) supplemented with 10% DMSO (Sigma Aldrich, MO, USA), aliquoted into cryovials, and frozen in a controlled-rate freezing container (Cryo 1 °C Freezing Container, Nalgene, NY, USA) at –80 °C overnight before being transferred to liquid nitrogen for long-term storage.

### Recall stimulation of PBMCs

For functional assays, frozen PBMCs were thawed in a 37 °C water bath and transferred into 20 mL of RPMI 1640 medium with GlutaMAX (Gibco, MA, USA). After centrifugation (200 × *g*, 10 min) and washing with 40 mL of RPMI, the cells were resuspended in complete medium consisting of RPMI supplemented with 10% human serum and 10 µg/mL of gentamicin. Following a 2-h resting period at 37°C in 5% CO_2_, viable cells were counted and numbers were adjusted before analyses. For flow cytometry, 8 × 10^5^ cells were plated per well in round-bottom 96-well plates (Sarstedt, Germany). For proliferation assays, 2 × 10^5^ cells were seeded per well. Selected wells were stimulated with Ft antigen [2.5 µg/mL; prepared from *F. tularensis* LVS as previously described (19)] or concanavalin A (ConA, 2.5 µg/mL). After 3 days, cells were prepared for flow cytometric analysis. Supernatants from parallel wells were collected and stored at –80 °C for cytokine measurements. Prior to stimulation and seeding, cells for lymphocyte proliferation assays were labeled with CellTrace Far Red according to the manufacturer’s instructions (Thermo Fisher Scientific, OR, USA). Four days later, cells were analyzed by flow cytometry to determine the proportion of proliferating cells. Each sample was analyzed in triplicates.

### Flow cytometry

After 72 h of recall stimulation, cells were incubated with Brefeldin A (5 µg/mL) and Monensin (5 µg/mL) for 4 h. Cells were then pelleted (500 × *g*, 3 min), and supernatants were discarded. Viability was assessed using Aqua Viability Dye (Invitrogen) for 20 min at room temperature, followed by staining with fluorochrome-conjugated monoclonal antibodies against surface markers (30 min at 4 °C). After washing and permeabilization (BD Biosciences fix/perm buffer, 20 min at 4 °C), cells were stained intracellularly for cytokines. The following antibody conjugates were used (BD Biosciences): CD3-APCH7 (SK7), CD4-BUV496 (SK3), CD8-BUV395 (RPA-T8), CD45RO-APC (UCHL-1), CCR7-Bv421 (2-L1-A), CD28-BB515 (CD28.2), CD95-R718 (DX2), IFNγ-BB700 (B27), MIP-1β-PE (D21-1351), IL-2-BV711 (5344.111), and TNF-BV650 (MAb11). Dump channel included CD14-V500 (M5E2) and CD19-V500 (HIB19). Samples were acquired using a ZE5 flow cytometer (Bio-Rad) and analyzed with FlowJo software (BD Biosciences). Each experimental setup included either samples from a single vaccinated donor collected at 1, 2, 4, and 12 weeks post-vaccination or PBMCs from two to three individuals obtained 1 and 12 months after onset of tularemia. As controls, each experiment also included PBMCs from a naïve donor and from a high-responder donor. Each sample was analyzed in duplicates or triplicates.

### Multiplex cytokine assay

Supernatants (50 µL/well) collected after 72 h of re-call stimulation were stored at –80 °C until analysis. Cytokine concentrations were quantified using a 17-plex kit (Bio-Rad Laboratories Inc., Hercules, CA, USA, M5000031YV) following the manufacturer’s instructions on a Bio-Plex 200 system (Bio-Rad).

### Multivariate analysis

Cytokine concentrations obtained from multiplex cytokine assay analysis of supernatants from recall-stimulated PBMCs were analyzed. The dataset included 16 cytokines (IL-1β, IL-2, IL-4, IL-5, IL-6, IL-7, IL-10, IL-12p70, IL-13, IL-17, G-CSF, GM-CSF, IFN-γ, MCP-1, MIP-1β, and TNF) measured in seven experimental groups (naïve controls, vaccinees at 1, 2, 4, and 12 weeks post-vaccination, and patients at 1 and 12 months post-infection). A total of 213 observations were included in principal component analysis (PCA). Factors were extracted using Varimax rotation with Kaiser normalization and a Kaiser criterion (Eigenvalue > 1). Sampling adequacy was confirmed by a Kaiser–Meyer–Olkin value of 0.889 and a significant Bartlett’s test of sphericity (*p <* 0.001).

Linear discriminant analysis (LDA) was conducted with the stepwise method based on Wilks’ Lambda applied with entry/removal criteria *F*_in_ = 3.84 and *F*_out_ = 2.71. Prior probabilities were based on group sizes, and Box’s *M* test was used to check homogeneity of covariance matrices. Classification accuracy was assessed using cross-validation (leave-one-out method).

### Statistical significance

Independent-samples Kruskal–Wallis test in SPSS was used to determine significant difference among groups. Pairwise *post-hoc* comparisons were adjusted by the Bonferroni correction. A *p-*value < 0.05 was considered statistically significant for all tests. All analyses were performed in IBM SPSS Statistics v29 (IBM Corp., Armonk, NY, USA).

## Results

### Proliferative responses of PBMCs

**Figure 1:**
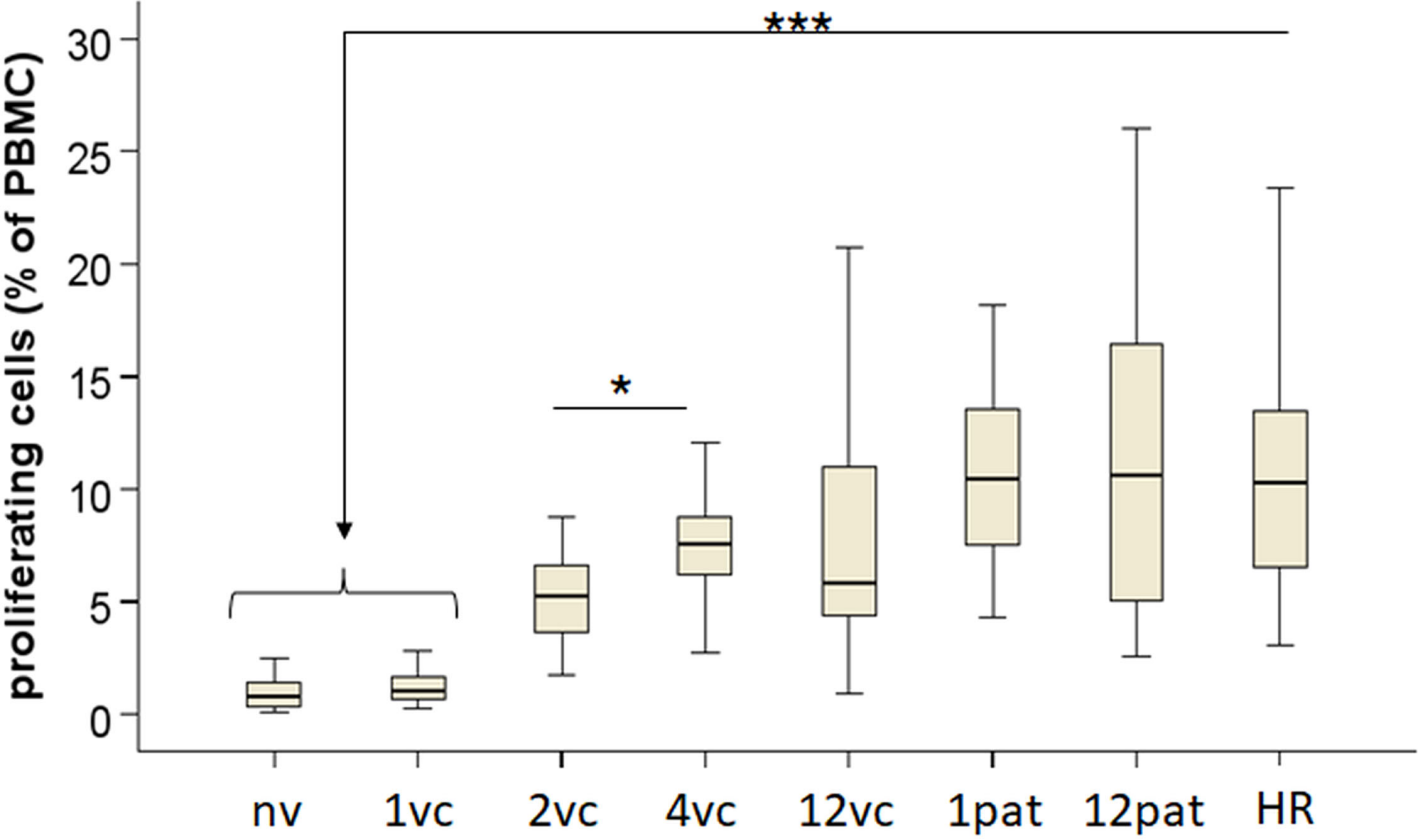
Proliferative responses of PBMC. PBMCs were labeled with CellTrace Far Red, stimulated with *F. tularensis* antigen for 4 days, and analyzed by flow cytometry to determine the proportion of proliferating cells. Samples were obtained from naïve donors, vaccinated donors at 1 (1vc), 2 (2vc), 4 (4vc), and 12 weeks (12vc) post-vaccination, or from patients with tularemia sampled 1 month (1pat) or 12 months (12pat) after onset. High responders (HR) represent PBMC from donors with verified strong immune responses to *F. tularensis*. Statistical comparisons were performed using the independent-samples Kruskal–Wallis test with Bonferroni correction. Asterisks indicate significant differences relative to naïve donors (*p < 0.05, ***p < 0.001).

PBMCs were stimulated with *F. tularensis* antigen for 4 days and analyzed by flow cytometry. The naïve group showed minimal or no proliferation, whereas vaccinees developed detectable responses from 2 weeks post-vaccination onwards (*p <* 0.001; **Figure 1**). High responders and patients displayed robust proliferation, significantly exceeding that of the naïve group (*p <* 0.001), whereas the two latter groups were indistinguishable (*p* > 0.05; **Figure 1**). Thus, vaccination and infection induced long-lived memory T cells as evidenced by strong proliferative responses observed at all time points investigated, except the 1-week time point.

### Detection of secreted cytokines and identification of cytokine patterns

Cytokine secretion from PBMCs stimulated by *F. tularensis* antigen for 3 days was determined. Cytokine secretion significantly higher than that of the naïve group was observed in samples from vaccinees and patients at all time points, except the 1- or 2-week time point samples post-vaccination (**Table 1**). For most cytokines, the concentrations were not significantly different between 1- and 12-month patient samples and 4- and 12-week vaccinee samples (**Table 1**, **Supplementary Table 1**). IL-4 and IL-5 levels were exceptions, as these were elevated in patient samples but not in vaccinee samples (**Table 1**, **Supplementary Table 1**).

**Table 1:** Cytokine concentrations in culture media after 3 days of stimulation of PBMC with *F. tularensis* antigen.

Cytokines	Status							
	Naïve^1^ (36)^2^	1vc^3^ (24)	2vc (24)	4vc (24)	12vc (24)	1pat^4^ (18)	12pat (33)	HR^5^ (30)
IL-1β	2.66 (0.48)^6^	2.60 (0.65)	2.81 (0.81)	2.87 (0.62)	2.79 (0.58)	2.82 (0.25)	2.57 (0.38)	2.70 (0.37)
IL-2	1.44 (0.19)	1.37 (0.36)	1.54 (0.32)	1.70 (0.23)***^7^	1.71 (0.24)**	1.76 (0.22)***	1.66 (0.15)**	1.67 (0.47)***
IL-4	1.67 (0.25)	1.62 (0.34)	1.74 (0.57)	1.85 (0.41)	1.81 (0.27)	1.96 (0.16)***	1.84 (0.13)**	1.83 (0.21)**
IL-5	2.72 (0.29)	2.68 (0.23)	2.74 (0.5)	2.84 (0.35)	2.81(0.32)	3.17 (0.24)***	3.02 (0.16)***	2.95 (0.33)***
IL-6	4.23 (0.45)	4.26 (0.55)	4.38 (0.84)	4.41(0.77)	4.37 (0.35)	4.41 (0.62)	4.29 (0.40)	4.11 (0.36)
IL-7	1.90 (1.33)	1.91 (1.45)	2.17 (0.75)	2.45 (0.34)*	2.35 (0.45)	2.73 (0.35)***	2.18 (0.71)	2.36 (0.53)**
IL-10	1.35 (0.63)	1.66 (0.49)	1.77 (0.53)	1.72 (0.38)**	1.77 (0.45)**	1.72 (0.30)**	1.63 (0.34)	1.63 (0.26)
IL-12p70	1.56 (0.74)	1.56 (0.39)	1.73 (0.42)	1.87 (0.23)**	1.77 (0.33)	2.08 (0.22)***	1.78 (0.46)	1.86 (0.31)***
IL-13	0.42 (0.72)	0.62 (0.62)	0.96 (0.60)**	1.32 (0.41)***	1.17 (0.56)***	1.32 (0.43)***	1.25 (0.63)***	1.07 (0.45)***
IL-17	1.96 (0.32)	1.98 (0.39)	2.13 (0.58)	2.26 (0.39)***	2.27 (0.38)***	2.36 (0.21)***	2.17 (0.25)*	2.24 (0.24)***
G-CSF	3.06 (0.37)	3.00 (0.83)	3.10 (1.14)	3.27 (1.01)	3.26 (0.82)	3.19 (0.49)	3.26 (0.47)	2.96 (0.33)
GM-CSF	2.07 (0.42)	1.94 (0.44)	1.95 (0.50)	1.99 (0.33)	2.10 (0.51)	2.38 (0.43)*	2.03 (0.38)	1.95 (0.47)
IFN-γ	2.51 (0.43)	2.51 (0.53)	3.03 (0.82)	3.39 (0.59)***	3.30 (0.44)**	3.81 (0.36)***	3.72 (0.92)***	4.22 (0.69)***
MCP-1	3.75 (0.39)	3.77 (0.34	3.79 (0.38)	3.83 (0.29)	3.75 (0.40)	3.81 (0.60)	3.96 (0.28)**	3.71 (0.34)
MIP-1β	3.75 (0.17)	3.72 (0.26)	3.74 (0.35)	3.77 (0.26)	3.79 (0.26)	3.85 (0.22)	3.87 (0.24)*	3.89 (0.24)**
TNF	4.04 (0.28)	3.94 (0.34)	4.15 (0.38)	4.32 (0.28)**	4.19 (0.22)	4.12 (0.21)	4.02 (0.25)	4.18 (0.31)

^1^ PBMC from individuals not vaccinated with *F. tularensis* LVS and with no history of tularemia.

^2^ Number of samples in the analysis.

^3^ PBMC from individuals 1 week after vaccination with *F. tularensis* LVS.

^4^ Patients had onset of tularemia approximately 1 month before collection of PBMC.

^5^ High responders, PBMC from individuals vaccinated with LVS and with a previously verified high recall response to *F. tularensis* antigen.

^6^ Median cytokine concentration (log_10_ pg/mL) and interquartile range (Q3–Q1) inside brackets.

^7^ Statistical differences among groups were evaluated with independent-samples Kruskal–Wallis test with Bonferroni correction for multiple tests. The median value of each group is compared to the median value of the naïve group. (*P < 0.05, **P < 0.01, ***P < 0.001).

Several cytokines were highly correlated (**Supplementary Table 2**), and PCA of the cytokine levels identified four latent factors explaining 78.4% of the total variance. Factor 1 accounted for 42.1% of the total variance and showed strong positive loadings (>0.7) for G-CSF, IL-6, IL-4, IL-1β, MCP-1, MIP-1β, and TNF (**Supplementary Table 3**). Factor 2 (F2) (17.2% of variance) was dominated by IFN-γ plus IL-13, whereas Factors 3 (F3) (11.4%) and 4 (6.5%) were dominated by IL-12p70 plus IL-10 and IL-7, respectively (**Supplementary Table 3**).

PCA-derived factor scores were analyzed using the Kruskal–Wallis test to assess differences between groups. Significant group differences were detected for the IFN-γ/IL-13-dominated Factor 2 (*p <* 0.001). Compared with the naïve group, factor scores were significantly higher in the 4- and 12-week vaccinee samples and in the 1- and 12-month patient samples (*p <* 0.001). The cytokine levels in the 1- and 2-week vaccinee samples did not differ from the naïve group samples (*p* > 0.05), and the two former groups showed significantly lower factor scores relative to all other groups (*p <* 0.001). Factor 2 scores were similar among the 4- and 12-week vaccinee samples, the 1- and 12-month patient samples, and the high responder samples (*p* > 0.05).

The PCA factors were evaluated using LDA to assess their ability to discriminate between groups. Two significant discriminant functions were extracted (Wilks’ λ = 0.336, *p <* 0.001). Function 1, (0.986F2) + (0.07F3), accounted for 93.7% of the discriminating power and was mainly associated with the IFN-γ/IL-13-dominated Factor 2. Function 2, (−0.167F2) + (0.998F3), contributed modestly (6.3%) and was strongly influenced by the IL-12p70/IL-10-dominated Factor 3 (−0.167F2) + 0.998F3).

The overall classification accuracy of the model was 32.4% and 29.6% as determined by cross-validation, suggesting limited discriminatory power. Cross-validation showed that 1-week vaccinee samples often were misclassified as naïve samples, and vice versa, reflecting minimal immune activation 1 week post-vaccination (**Table 2**). Misclassification decreased at later time points, and 4- and 12-week vaccinee samples, 1- and 12-month patient samples, and high responder samples were rarely classified as naïve (0%–9%), indicating distinct cytokine profiles (**Table 2**). However, misclassifications between these groups of samples from immune individuals were frequent.

**Table 2:** Classification accuracy of LDA-generated model based on function 1^1^ and 2^2^ assessed using cross-validation, leave-one-out method.

	Predicted groups (%)							
	Nv^3^	1vc^4^	2vc	4vc	12vc	1pat^5^	12pat	HR^6^
Naïve	55.6	25.0	5.6	0.0	8.3	0.0	5.6	0.0
1vc	50.0	25.0	20.8	0.0	0.0	0.0	4.2	0.0
2vc	20.8	8.3	29.2	16.7	16.7	0.0	4.2	4.2
4vc	4.2	0.0	25.0	16.7	16.7	0.0	25.0	12.5
12vc	4.2	4.2	25.0	25.0	8.3	0.0	20.8	12.5
1pat	0.0	0.0	0.0	27.8	11.1	0.0	50.0	11.1
12pat	9.1	0.0	15.2	3.0	12.1	0.0	21.2	39.4

^1^ Function 1: (0.986*Factor 2) + (0.007*Factor 3).

^2^ Function 2: (−0.167*Factor 2) + (0.998 *Factor 3).

^3^ Naïve.

^4^ PBMC from individuals 1 week after vaccination with *F. tularensis* LVS.

^5^ Patient had onset of tularemia approximately 1 month before collection of PBMC.

^6^ High responders, PBMC from individuals vaccinated with LVS and with a previously verified high recall response to *F. tularensis* antigen.

In summary, Function 2, predominantly based on Factor 2 (IFN-γ and IL-13), distinguished samples from immune and naïve individuals, although the model lacked sufficient resolution to separate the samples from different groups of immune individuals.

### Detection of intracellular cytokines

**Figure 2:**
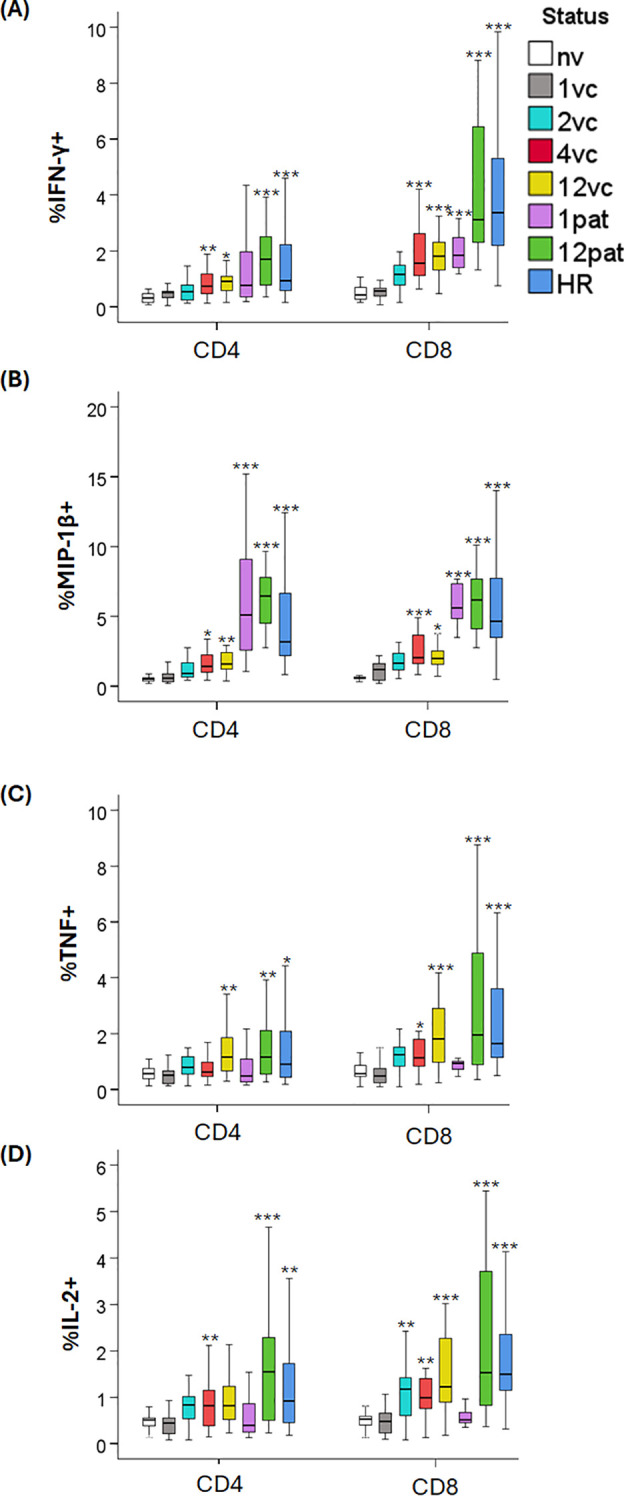
Intracellular cytokine expression by CD4 and CD8 T cells. PBMCs were stimulated with *F. tularensis* antigen for 3 days and thereafter stained with a panel of antibodies to determine the frequency of CD4 and CD8 T cells expressing IFN-γ **(A)**, MIP-1β **(B)**, TNF **(C)**, and IL-2, (**D** respectively). Samples were obtained from naïve donors (nv), vaccinated donors at 1 (1vc), 2 (2vc), 4 (4vc), and 12 weeks (12vc) post-vaccination, or from patients with tularemia sampled 1 month (1pat) or 12 months (12pat) after onset of disease. High responders (HR) represented PBMC from vaccinated donors with previously verified strong immune responses to *F. tularensis*. Statistical comparisons were performed using the independent-samples Kruskal–Wallis test with Bonferroni correction. Asterisks indicate significant differences relative to naïve donors (*p < 0.05, **p < 0.01, ***p < 0.001).

To further characterize T-cell responses following *F. tularensis* exposure, PBMCs were analyzed for intracellular cytokine expression after 3 days of antigen stimulation (**Figure 2**). Samples from the naïve group exhibited minimal or no cytokine expression of all cytokines tested. In contrast, samples from vaccinees showed significant increases of cytokine-producing cells from 4 weeks post-vaccination onwards. At weeks 4 and 12 post-vaccination, CD8 T cells displayed significantly higher frequencies of IFN-γ^+^, MIP-1β^+^, TNF^+^, and IL-2^+^ cells compared with the naïve group (*p <* 0.05–0.001; **Figures 2A–D**). A similar increase was detected in CD4 T cells with the exception for TNF and IL-2 at 4 weeks post-vaccination (*p <* 0.05–0.01; **Figures 2A–D**).

In samples from patients 1 month post-infection, CD4 and CD8 T cells expressing MIP-1β as well as CD8 T cells expressing IFN-γ were significantly increased relative to the naïve group (*p <* 0.001; **Figures 2A–D**). Samples obtained 12 months post-infection exhibited strong and consistent increases of CD4 and CD8 T cells expressing IFN-γ, MIP-1β, TNF, and IL-2 (*p <* 0.01–0.001; **Figures 2A–D**). These cytokine profiles closely resembled those of the high responders (**Figures 2A–D**).

The most notable differences between vaccinee and patient samples were the higher frequencies of MIP-1β^+^ CD4 and CD8 T cells in patient samples at the 1- and 12-month time points (*p <* 0.05–0.001; **Table 3**; **Figures 2A–D**). In addition, IFN-γ^+^ CD8 T cells were more frequent in patient samples at the 12-month time point (*p <* 0.05–0.001; **Table 3**; **Figures 2A–D**). In one instance, cytokine expression was elevated in samples from vaccinated individuals compared to patients, as higher frequencies of IL-2^+^ CD4^+^ and CD8^+^ T cells were detected in the 4-week samples from vaccinees vs. the 1-month patient samples (**Figure 2D**).

**Table 3:** Statistical comparisons of frequencies of cytokine-expressing CD4 or CD8 cells between patients and vaccinees.

		CD4				CD8			
S1^1^	S2	IFN-γ	MIP-1B	TNF	IL-2	IFN-γ	MIP-1B	TNF	IL-2
1pat^2^	Naïve	–	<0.001^3^	–	–	<0.001	<0.001	–	–
	1vc^4^	<0.001	<0.001	–	–	<0.001	<0.001	–	–
	2vc	–	<0.001	–	–		<0.001	–	–
	4vc	–	<0.001	–	–		<0.05	–	–
	12vc	–	0.011	–	–		<0.01	–	–
	12pat	–	–	–	–		–	–	<0.01
	HR	–	–	–	–		–	–	–
12pat	Naïve	<0.001	<0.001	<0.01	<0.001	<0.001	<0.001	<0.001	<0.001
	1vc	<0.001	<0.001	<0.001	<0.001	<0.001	<0.001	<0.001	<0.001
	2vc	<0.001	<0.001	–	–	<0.001	<0.001	–	–
	4vc	–	<0.001	–	–	<0.05	<0.01	–	–
	12vc	–	<0.001	–	–	<0.01	<0.001	–	–
	1pat	–	–	–	<0.01	–	–	–	<0.01
	HR	–	–	–	<0.01	–	–	–	–

^1^ Each row tests the null hypothesis that the Sample 1 (S1) and Sample 2 (S2) distributions are the same.

^2^ PBMC sampled from patients 1 month postinfection.

^3^ *p*-value.

^4^ PBMC sampled 1 week postinfection.

In summary, these results demonstrate that vaccination elicited immunospecific cytokine-expressing CD4 and CD8 T cells from 4 weeks onwards and that natural infection induced qualitatively similar but quantitatively stronger responses that persisted for at least 1 year.

### Intracellular cytokine responses in the CD4 memory subpopulations

**Figure 3:**
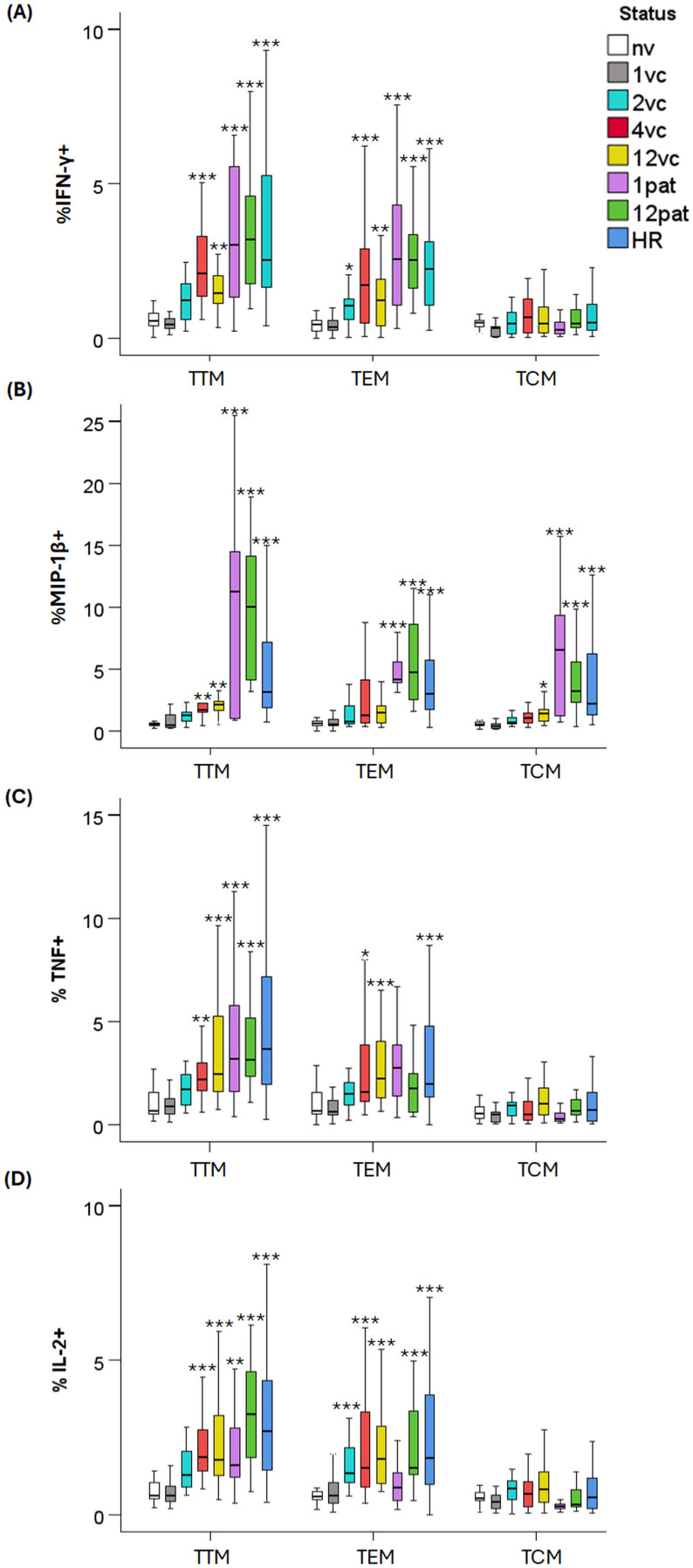
Intracellular cytokine expression by CD4 subsets. PBMCs were stimulated with *F. tularensis* antigen for 3 days and thereafter stained with a panel of antibodies to determine the frequency of CD4/TTM, CD4/TEM, and CD4/TCM cells expressing IFN-γ **(A)**, MIP-1β **(B)**, TNF **(C)**, and IL-2, (**D** respectively). Samples were obtained from naïve donors (nv), vaccinated donors at 1 (1vc), 2 (2vc), 4 (4vc), and 12 weeks (12vc) post-vaccination, or from patients with tularemia sampled 1 month (1pat) or 12 months (12pat) after onset of disease. High responders (HR) represented PBMC from vaccinated donors with previously verified strong immune responses to *F. tularensis*. Statistical comparisons were performed using the independent-samples Kruskal–Wallis test with Bonferroni correction. Asterisks indicate significant differences relative to the naïve group (* *p <* 0.05, **p < 0.01 and ***p < 0.001).

The frequencies of CD4 transitional memory T cells (TTM) expressing either IFN-γ, MIP-1β, TNF, or IL-2 were elevated in vaccinee samples relative to the naïve group from 4 weeks onwards and similarly elevated numbers were observed in samples from patients and high responders (*p <* 0.05–0.001; **Figure 3**). Also, cytokine expressing CD4 effector memory T cells (TEM) showed elevated frequencies in the same samples, except that MIP-1β expressing TEM was not elevated in the 4- and 12-week samples from vaccinees (*p <* 0.05–0.001; **Figure 3**). Frequency of CD4 central memory T cells (TCM) expressing MIP-1β was elevated in 12-week vaccine samples and in 1- and 12-month patient samples; however, no increase was detected for this and the other cytokines analyzed (*p <* 0.05–0.001; **Figure 3**).

T stem cell memory (TSCM) cells are long-lived and represent the earliest, most multipotent memory T-cell subset. The frequency of such cytokine-expressing cells was low and comparable in samples from vaccinees, patients, and the naïve group (**Supplementary Figure 2**). MIP-1β^+^ TSCM cells were an exception, with significantly elevated frequencies in samples from patients at 1 and 12 months post-infection and in high responders relative to those of naïve individuals (*p <* 0.001; **Supplementary Figure 2**).

The most notable differences were the higher frequencies of CD4 TEM, TTM, and TSCM cells expressing MIP-1β in patient samples compared with those from vaccinees (*p <* 0.05–0.001; **Table 4**; **Figure 3**; **Supplementary Figure 2**). Differences were also observed for the other cytokines; in the majority of cases, frequencies were lower in samples collected 1 and 2 weeks post-vaccination but levels in samples from later time points were similar to those in patient samples at 1 and 12 months post-infection (*p <* 0.05–0.001; **Table 4**; **Figure 3**; **Supplementary Figure 2**).

**Table 4:** Statistical comparisons of frequencies of cytokine-expressing CD4 memory populations between patients and vaccinees.

		CD4/TTM				CD4/TEM			
S1^1^	S2	IFN-γ	MIP-1B	TNF	IL-2	IFN-γ	MIP-1B	TNF	IL-2
1pat^2^	Naïve	<0.001^3^	<0.001	<0.001	<0.01	<0.001	<0.001	<0.01	–
	1vc^4^	<0.001	<0.001	<0.01	<0.01	<0.001	<0.001	<0.01	–
	2vc	–	<0.01	–	–	–	<0.001	–	–
	4vc	–	–	–	–	–	<0.05	–	–
	12vc	–	–	–	–	–	<0.01	–	–
	12tul	–	–	–	–	–	–	–	<0.05
	HR	–	–	–	–	–	–	–	–
12pat	Naïve	<0.001	<0.001	<0.001	<0.001	<0.001	<0.001	–	–
	1vc	<0.001	<0.001	<0.001	<0.001	<0.001	<0.001	–	–
	2vc	<0.001	<0.001	<0.05	<0.01	<0.01	<0.001	–	–
	4vc	–	<0.001	–	–	–	<0.01	–	–
	12vc	–	<0.001	–	–	<0.01	<0.001	–	–
	1tul	–	–	–	–	–	–	–	–
	HR	–	–	–	–	–	–	–	

^1^ Each row tests the null hypothesis that the Sample 1 (S1) and Sample 2 (S2) distributions are the same.

^2^ PBMC sampled from patients 1 month postinfection.

^3^ *p*-value.

^4^ PBMC sampled 1 week postinfection.

In summary, antigen-specific cytokine production was most marked for the CD4 TTM and TEM cell subsets, while TCM cells contributed modestly. MIP-1β^+^ responses of CD4 TTM, TEM, and TSCM cells distinguished the patient group from the vaccinee group, with the highest values in the former group.

### Intracellular cytokine responses in the CD8 memory populations

The frequency of cytokine-expressing CD8 T naïve (TNV) and TSCM cells was low and comparable in the samples from naïve individuals, vaccinees, and patients, with a few exceptions shown in **Supplementary Figure 3**.

**Figure 4:**
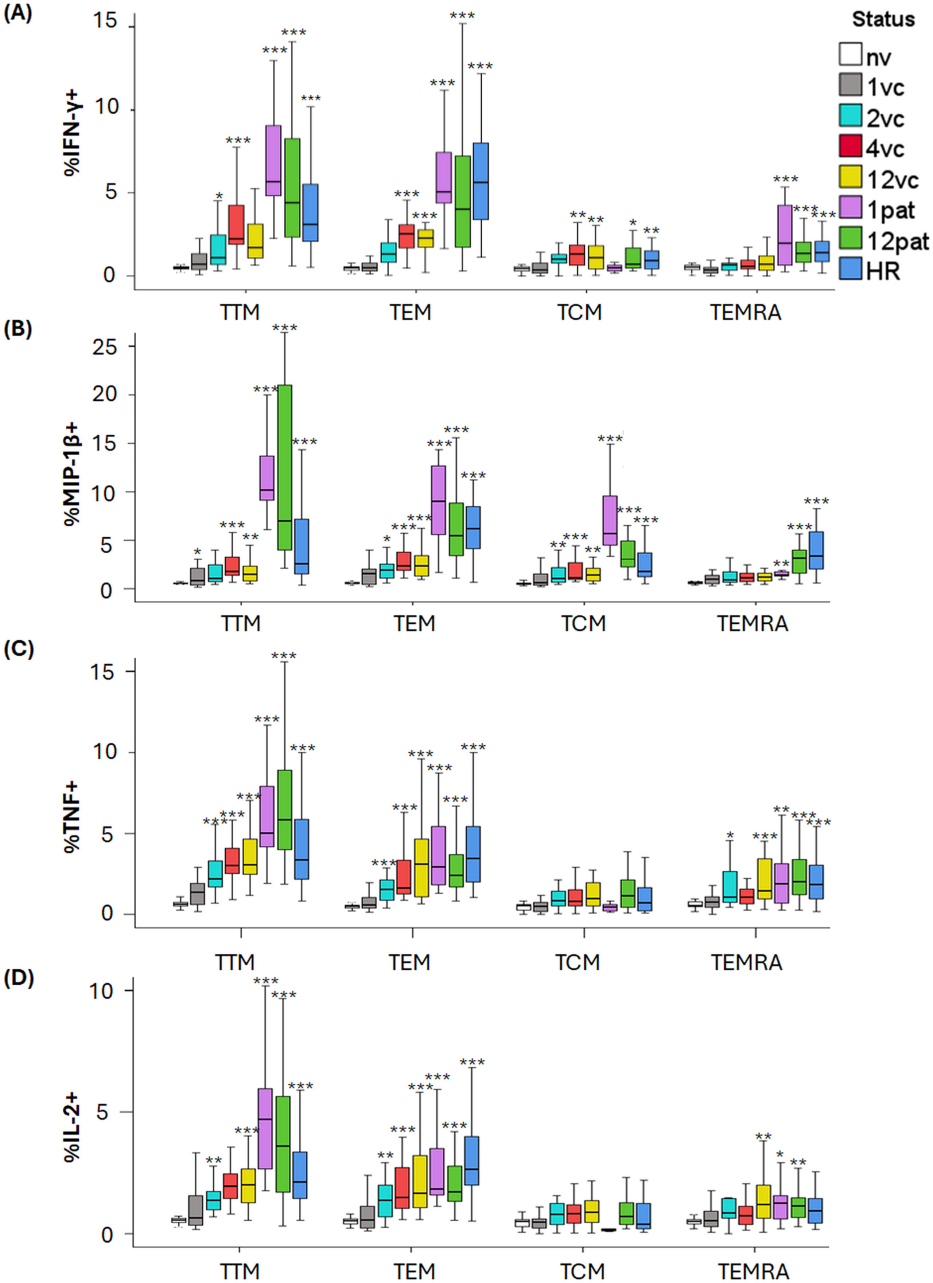
Intracellular cytokine expression by CD8 subsets. PBMCs were stimulated with *F. tularensis* antigen for 3 days and thereafter stained with a panel of antibodies to determine the frequency of the memory populations expressing IFN-γ **(A)**, MIP-1β **(B)**, TNF **(C)**, and IL-2, (**D** respectively). Samples were obtained from naïve donors (nv), vaccinated donors at 1 (1vc), 2 (2vc), 4 (4vc), and 12 weeks (12vc) post-vaccination, or from patients with tularemia sampled 1 month (1pat) or 12 months (12pat) after onset of disease. High responders (HR) represented PBMC from vaccinated donors with previously verified strong immune responses to *F. tularensis*. Statistical comparisons were performed using the independent-samples Kruskal–Wallis test with Bonferroni correction. Asterisks indicate significant differences relative to the naïve group (*P < 0.05, **P < 0.01, ***P < 0.001).

Cytokine-expressing CD8 memory T cells were detected among the TTM, TEM, TCM, and TEMRA (Terminal Effector Memory T cells re-expressing CD45RA) populations (**Figures 4A–D**). Compared with the naïve group, the frequencies of IFN-γ^+^, MIP-1β^+^, TNF^+^, and IL-2^+^ cells were significantly increased in the TTM and TEM populations in most samples from vaccinees at 2 weeks onwards, as well as in all samples from patients and high responders (*p <* 0.01–0.001; **Figures 4A, B**). A similar pattern was observed for IFN-γ^+^ and MIP-1β^+^ TCM cells (*p <* 0.01–0.001; **Figures 4A, B**).

The frequencies of IFN-γ^+^, MIP-1β^+^, TNF^+^, and IL-2^+^ TEMRA cells were significantly elevated in samples from patients and high responders vs. the naïve group (*p <* 0.05–0.001; **Figures 4A, B**). In contrast, TEMRA responses in samples from vaccinees were modest and only higher frequencies of TNF^+^ and IL-2^+^ cells at 12 weeks post-vaccination discriminated these samples from those of the naïve group (*p <* 0.01–0.001; **Figures 4A, B**).

The most notable differences between vaccinees and patient samples were higher frequencies of MIP-1β+ TTM, TEM, and TEMRA cells for most time points (*p <* 0.05–0.001; **Table 5**; **Figures 4A–D**). In addition, samples from patients and vaccinees were differentiated regarding frequencies of IFN-γ among the TEM and TEMRA subsets, again levels being higher in the patient group for most time points (*p <* 0.05–0.01; **Table 4**; **Figure 4A**).

**Table 5:** Statistical comparisons of frequencies of cytokine-expressing CD8 memory populations between patients and vaccinees.

		CD8/TTM				CD8/TEM				CD8/TCM				CD8/TEMRA			
S1^1^	S2	IFN-γ	MIP-1B	TNF	IL-2	IFN-γ	MIP-1B	TNF	IL-2	IFN-γ	MIP-1B	TNF	IL-2	IFN-γ	MIP-1B	TNF	IL-2
1pat^2^	Naïve	–	<0.001^3^	–	–	<0.001	<0.001	<0.001	<0.001	–	<0.001^3^	–	–	<0.001	<0.01	<0.01	<0.05
	1vc^4^	–	<0.001	–	–	<0.001	<0.001	<0.001	<0.001	–	<0.001	–	–	<0.001	–	–	–
	2vc	–	<0.001	–	<0.01	<0.001	<0.001	–	–	–	<0.001	–	<0.01	<0.05	–	–	–
	4vc	<0.05	<0.001	–	<0.01	–	<0.05	–	–	<0.05	<0.001	–	<0.01	<0.05	–	–	–
	12vc	–	<0.001	–	<0.001	<0.05	<0.01	–	–	–	<0.001	–	<0.001	–	–	–	–
	12pat	–	–	<0.05	<0.01	–	–	–	–	–	–	<0.05	<0.01	–	–	–	–
	HR	–	<0.01	–	0.008	–	–	–	–	–	<0.01	–	0.008	–	–	–	–
12pat	Naïve	<0.05	<0.001	<0.05	–	<0.001	<0.001	<0.001	<0.001	<0.05	<0.001	<0.05	–	<0.001	<0.01	<0.001	<0.01
	1vc	<0.05	<0.001	<0.05	–	<0.001	<0.001	<0.001	<0.001	<0.05	<0.001	<0.05	–	<0.001	<0.01	<0.001	<0.05
	2vc	–	<0.001	–	–	<0.01	<0.001	–	–	–	<0.001	–	–	<0.01	<0.01	–	–
	4vc	–	<0.05	–	–	–	–	–	–	–	<0.05	–	–	<0.01	<0.05	–	–
	12vc	–	<0.01	–	–	–	<0.01	–	–	–	<0.01	–	–	<0.05	<0.05	–	–
	HR	–	<0.01	–	–	–	–	–	–	–	–	–	–	–	–	–	–

^1^ Each row tests the null hypothesis that the Sample 1 (S1) and Sample 2 (S2) distributions are the same.

^2^ PBMC sampled from patients 1 month postinfection.

^3^ *p*-value.

^4^ PBMC sampled 1 week postvaccination.

## Discussion

Memory cells constitute the basis for cellular immunity. Even though immunity may last for several decades, or even be lifelong, the usual memory cells may be rather short-lived, typically in the order of months (20). The paradox is resolved by the presence of clonal populations of memory cells with characteristic phenotypes that are sustained and thereby achieve longevity of CMI. Therefore, characterization of individual T cells in immune individuals, serving as representatives of such clonal populations, will provide information essential to understanding how memory CMI is sustained and protective immune responses are achieved.

Collectively, our study provides a comprehensive and nuanced picture of the immunoreactivity following tularemia or tularemia vaccination. There were substantial commonalities, as proliferation assays, cytokine secretion profiles, and intracellular cytokine analyses consistently demonstrated that both exposures elicited robust, antigen-specific T-cell activation. In addition, analyses of secreted cytokines showed that proliferative responses were accompanied by coordinated cytokine release, while intracellular staining identified the responding cells as multifunctional memory CD4 and CD8 T cells.

PBMC proliferation data clearly discriminated naïve and immune individuals, in as much as vaccinees developed detectable proliferative responses from 2 weeks post-vaccination onwards and patients displayed robust responses at the time points investigated. The persistence of strong proliferative capacity in patients up to 12 months post-infection emphasizes the durability of the acquired cellular immunity to *F. tularensis*. This was not surprising, given that we previously demonstrated sustained cell-mediated immune responses for three decades after tularemia vaccination without evidence of decline (10, 11). Importantly, the marked similarities between high responders and patients suggest that a subset of vaccinated individuals can mount responses comparable in magnitude and quality to those induced by infection, supporting the immunogenic potential of the vaccine.

Analysis of secreted cytokines revealed a coordinated Th1-dominated cytokine response following vaccination with cytokine patterns clearly distinguished from those of naïve individuals emerging at 4 weeks post-vaccination. The convergence of cytokine patterns between vaccinees at these later time points and patients suggests that vaccination ultimately recapitulates major elements of infection-induced immunity. However, within this overall Th1-skewed milieu, a subset of cytokines classically associated with a Th2 immune response—IL-4, IL-5, IL-10, and IL-13—were selectively elevated in immune individuals. Rather than indicating a deviation from Th1-mediated immunity, these cytokines may reflect a broader immune response state involving regulatory mechanisms that fine-tune inflammation and support the transition from effector to memory phases, consistent with observations reported in other studies of immune memory (21, 22). For example, IL-4 and IL-5 can promote the induction of regulatory T cells that produce IL-10, an anti-inflammatory cytokine that contributes to the suppression of excessive Th1 responses and limits immunopathology. Notably, levels of IL-4 and IL-5 were selectively elevated in patient samples and in the control group high responders. The significant difference between the group of recent vaccinees and the high responders, also a group of vaccinees, is intriguing. Possibly, the high responder group consisted of individuals genetically predisposed to respond with vigorous IL-4 and IL-5 secretion upon this type of immune activation.

Logistical modeling, as illustrated by the LDA used in the present study, has been implemented in various forms in previous studies to delineate characteristics of the immune responses following tularemia vaccination. The parameters best predicting immune status have been linked to protective capacity (12, 13, 23–25). Herein, the multivariate analyses identified IFN-γ and IL-13 as central drivers of immune discrimination. Factor 2, dominated by these cytokines, consistently discriminated immune from naïve individuals and was significant in both vaccinees from 4 weeks post-vaccination and patients at all time points. While LDA confirmed the utility of this cytokine axis in distinguishing immune from naïve subjects, the limited classification accuracy highlights substantial overlap among immune groups, suggesting that vaccination and infection generate broadly similar cytokine milieus that are difficult to disentangle at the population level using soluble mediators alone.

The findings herein agree with our previous data demonstrating key roles of IFN-γ and IL-13 combined to identify the immune status following tularemia vaccination (12, 13). IFN-γ is well established as a key mediator of protection against intracellular pathogens such as *F. tularensis* (5, 26–29), and its prominence across analyses reinforces its role as a core correlate of cellular immunity in this setting. The contribution of IL-13 in the context of protection to an intracellular pathogen is less intuitive, as it is typically associated with Th2 immunity and M2 macrophage polarization, a phenotype that is generally less efficient than M1 macrophages in providing nitric oxide-mediated control of intracellular bacteria (30–32). Thus, rather than contributing directly to bacterial eradication, IL-13 is more likely to exert well-documented immunomodulatory and regulatory functions in concert with IL-4 and IL-10, and in processes related to tissue repair and resolution of inflammation (21, 22, 33).

Intracellular cytokine staining provided high-resolution insight into antigen-specific T-cell functionality. In agreement with previous findings, vaccination elicited significant increases in IFN-γ-, MIP-1β-, TNF-, and IL-2-expressing CD8 cells from 4 weeks post-vaccination onwards, with CD4 T-cell responses becoming more pronounced at later time points (12, 13, 34). Similarly, strong and polyfunctional cytokine profiles observed in patients, closely resembling those of high responders, indicate the establishment of a mature and durable memory T-cell compartment following both vaccination and infection. This prominent presence of IFN-γ-producing cells was not unexpected, since the critical role of the cytokine has been identified in numerous studies and elevated levels observed for more than three decades (11, 27, 28, 35–38). An intriguing finding was the elevated CD4 and CD8 T-cell expression of IL-2 in the 4-week vaccinee samples but lack thereof in the 1-month patient samples, despite the fact that levels of secreted cytokines were similar. Unlike other cytokines, the intracellular expression of IL-2 is highly dynamic, and it is exported quickly upon synthesis, but when bound to its receptor on the T cell, it is internalized and degraded. This highly dynamic process is critical for regulating proliferation. In view of the rather high levels of secreted IL-2, the low intracellular expression in the 1-month patient samples may therefore reflect a regulatory process subsequent to intense proliferation.

Across both CD4 and CD8 lineages, certain memory T-cell subsets were the primary contributors to cytokine production. Specifically, TTM and TEM cells consistently displayed the highest frequencies of cytokine-producing cells, whereas TCM and TSCM cells contributed modestly. The limited cytokine expression within TSCM populations is consistent with their early differentiation state and functional quiescence, although the selective enrichment of MIP-1β^+^ TSCM cells in patients suggests that natural infection may preferentially imprint chemokine-producing capacity even within long-lived memory pools. The TEM subpopulation is known to rapidly upregulate effector functions and to express homing receptors for migration to nonlymphoid sites of inflammation and to possess high levels of gut-homing molecules and chemokine receptors (39, 40). TTM cells display an intermediate phenotype between TEM and TCM subpopulations, since some phenotypic characteristics closely align with those of TEM cells, whereas others with those of TCM cells. Thus, the TEM and TTM populations represent potential effector populations and the identification of their potent upregulation of multiple cytokines is therefore logical and identify them as important for the effective protective responses occurring after vaccination or tularemia.

In the present study, individuals with a history of natural infection displayed not only a higher frequency of terminally differentiated TEMRA cells than the vaccinated group but also an increased proportion of MIP-1β-producing memory T-cell subsets, including the TTM, TEM, and TEMRA subsets. This pattern is consistent with a T-cell memory compartment characterized by enhanced effector and chemokine-producing capacity.

Immune senescence, which reflects the cumulative effects of aging and repeated antigen exposure, is commonly associated with a shift toward more differentiated and inflammatory T-cell phenotypes (41, 42). The higher mean age of the naturally infected cohort is therefore a relevant factor, as aging itself is linked to the accumulation of late-stage memory populations and increased baseline inflammatory activity. In this context, the elevated frequencies of MIP-1β-expressing memory T cells may reflect age-associated immune remodeling. However, a previous study of LVS vaccinees demonstrated that MIP-1β^+^ TEMRA cells were increased from 2 weeks onwards (12). Thus, vaccination induces MIP-1β^+^ TEMRA cells in certain individuals, as was also demonstrated in the high-responding group included in this study. A strength of the present study is the parallel analysis of samples from both vaccinees and patients. Moreover, natural infection is likely to provide a more intense and prolonged antigenic and inflammatory stimulus than vaccination, potentially promoting both advanced T-cell differentiation and sustained chemokine expression (43–45). The enrichment of MIP-1β-producing TTM, TEM, and TEMRA cells in patient samples may thus represent the combined effects of age-related immune senescence and infection-driven immune activation.

Studies on immunity after tularemia vaccination or human infection have identified multifunctional T cells similar to those described for patients with tuberculosis (46). When human, rat, or mouse co-culture systems have been used, correlations between levels of IFN-γ, TNF, and MIP-1β and protection were observed (12, 25, 47). Thus, there is substantial indirect evidence that levels of Th1 cytokines, such as IFN-γ, TNF, and MIP-1β, are correlates of protection in various animal and human tularemia models, thereby in much agreement with the present findings (5, 8).

A limitation of studies based on human samples is that immune cells are obtained almost exclusively from peripheral blood, which precludes evaluation of the protective role of tissue-resident cells. Animal models help address this gap; one commonly used model for tularemia is the mouse. Although not ideal—given the high susceptibility of mice to tularemia—it nevertheless has been extensively utilized. In a recent study employing parabiotic mice, optimal protection was found to depend on both tissue-resident and circulating T cells (48). In view of the effective protection conferred by the LVS vaccine, it is therefore possible that the vaccination primes both tissue-resident and circulating T cells in humans.

In summary, vaccination against *F. tularensis* induces robust, durable, and antigen-specific T-cell responses that mirror most aspects of natural infection, particularly with respect to IFN-γ-dominated immunity and general memory T-cell subset involvement. However, natural infection is associated with higher frequencies of MIP-1β-producing cells and more pronounced TEMRA responses, indicating qualitative differences in memory imprinting. These findings have important implications for the understanding of vaccine-mediated mechanisms, suggesting that while the LVS vaccine successfully elicits protective cellular immunity, it may not fully replicate the breadth and functional intensity of infection-induced T-cell responses.

## Data Availability

The original contributions presented in the study are included in the article/**Supplementary Material**. Further inquiries can be directed to the corresponding author.
