# Supplementary material for: Tularemia and tularemia vaccination induce similar, yet partially distinct, multifunctional T-cell responses
Source: Front Immunol. 2026 May 19;17:1812669. doi: 10.3389/fimmu.2026.1812669 (PMC13226113; doi:10.3389/fimmu.2026.1812669)
Supplement: Supplementary file 1 [file DataSheet1.docx]

**Supplementary data**

Figure. S1

**Figure S1. Illustration of gating strategy used to identify subpopulations in PBMC cells stained with** CD3-APCH7 (SK7), CD4-BUV496 (SK3), CD8-BUV395 (RPA-T8), CD45RO-APC (UCHL-1), CCR7-Bv421 (2-L1-A), CD28-BB515 (CD28.2), CD95-R718 (DX2), IFNγ-BB700 (B27), MIP-1β-PE (D21-1351), IL-2-BV711 (5344.111), TNF-BV650 (MAb11). Dump channel included CD14-V500 (M5E2) and CD19-V500 (HIB19). Samples were acquired using a ZE5 flow cytometer (BioRad) and analyzed with FlowJo software (BD Biosciences).

Figure S2


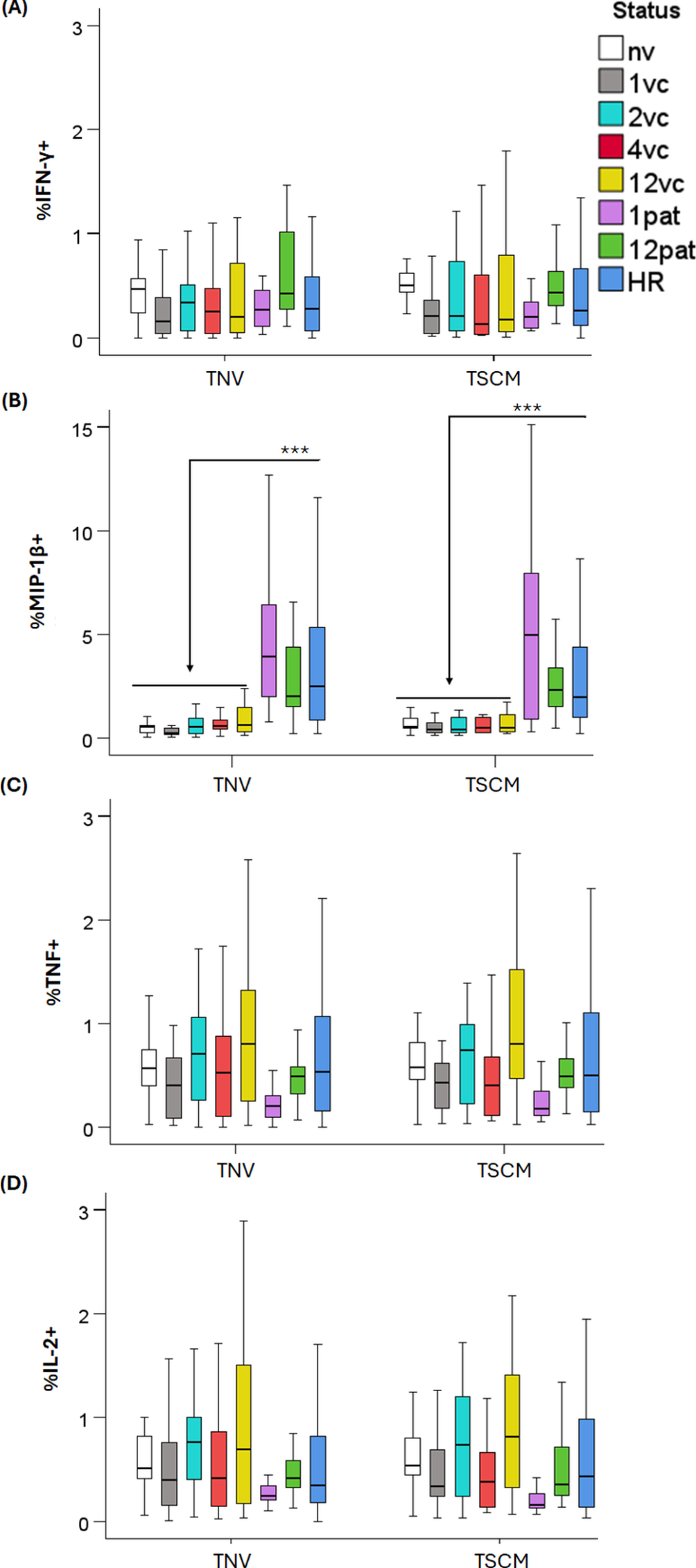


**Figure S2. Intracellular cytokine expression of CD4 populations.** PBMC were stimulated with *F. tularensis* antigen for three days and thereafter stained with a panel of antibodies to determine the frequency of CD4/TNV and CD4/TSCM cells expressing IFN-γ, MIP-1β, TNF, and IL-2, respectively. Samples were obtained from naïve donors (nv), vaccinated donors at 1 (1vc), 2 (2vc), 4 (4vc), and 12 weeks (12vc) post-vaccination, or from tularemia patients sampled 1 month (1pat) or 12 months (12pat) after onset of disease. High responders (HR) represented PBMC from vaccinated donors with previously verified strong immune responses to *F. tularensis*. Statistical comparisons were performed using the independent-samples Kruskal–Wallis test with Bonferroni correction. Asterisks indicate significant differences relative to indicated groups (****P <* 0.001).

Figure S3


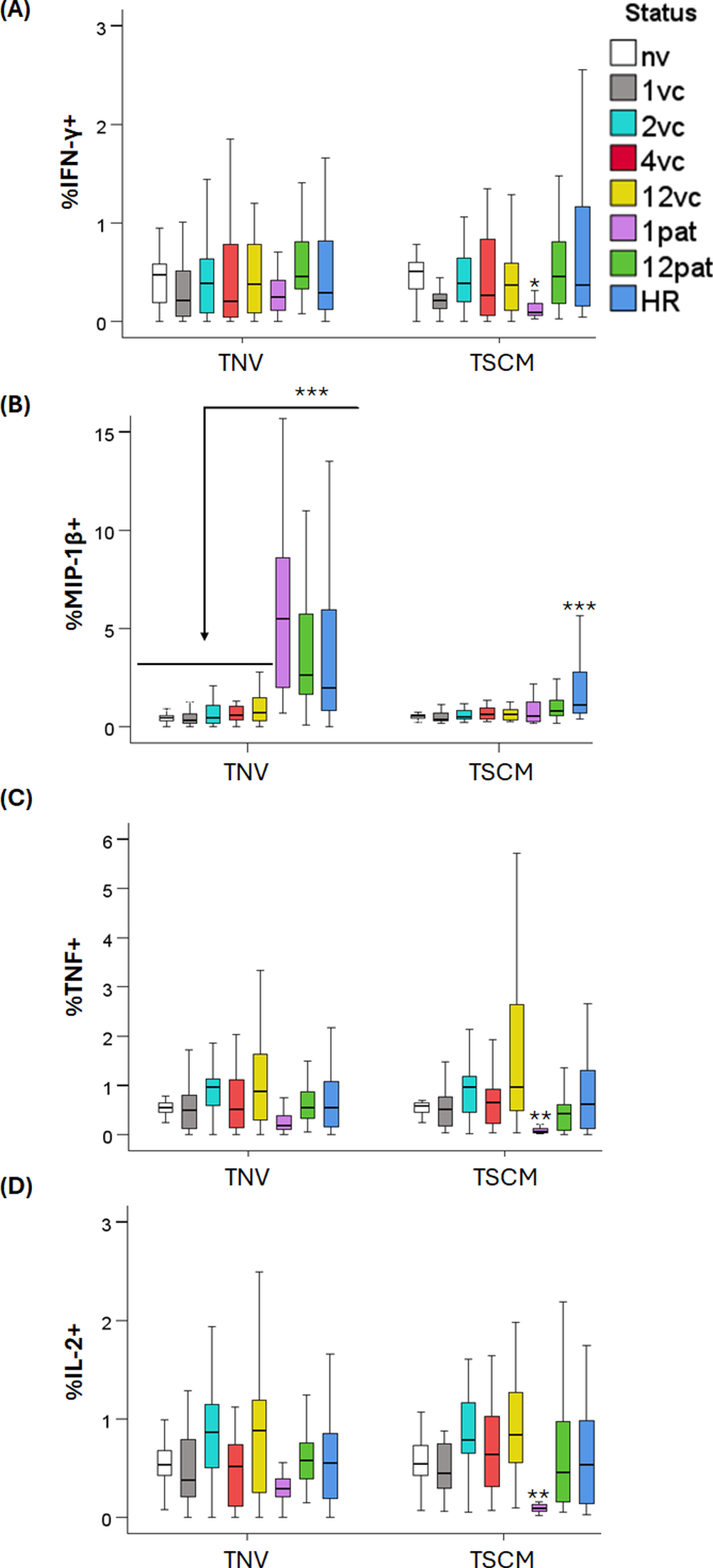


**Figure S3. Intracellular cytokine responses of CD8 populations.** PBMC were stimulated with *F. tularensis* antigen for three days and thereafter stained with a panel of antibodies to determine the frequency of CD8⁺/TNV and CD8/TSCM cells expressing IFN-γ, MIP-1β, TNF, and IL-2, respectively. Samples were obtained from naïve donors (nv), vaccinated donors at 1 (1vc), 2 (2vc), 4 (4vc), and 12 weeks (12vc) post-vaccination, or from tularemia patients sampled 1 month (1pat) or 12 months (12pat) after onset of disease. High responders (HR) represented PBMC from vaccinated donors with previously verified strong immune responses to *F. tularensis*. Statistical comparisons were performed using the independent-samples Kruskal–Wallis test with Bonferroni correction. Asterisks indicate significant differences relative to indicated groups (****P <* 0.001).

Table S1. Statistical comparisons of frequencies of secreted cytokines between patients and vaccinees.

| **Cytokines** | **1pat^1^ versus** | | |  | **12pat^2^ versus** | |
| --- | --- | --- | --- | --- | --- | --- |
|  | **4vc** | **12vc** | **12pat** |  | **4vc** | **12vc** |
| IL-1β | -^3^ | - | < 0.01^4^ |  | < 0.05 | - |
| IL-2 | - | - | - |  | - | - |
| IL-4 | - | < 0.05 | - |  | - | - |
| IL-5 | < 0.05 | < 0.001 | - |  | - | < 0.05 |
| IL-6 | - | - | - |  | - | - |
| IL-7 | - | - | - |  | - | - |
| IL-10 | - | - | - |  | - | - |
| IL-12p70 | - | - | < 0.01 |  | - | - |
| IL-13 | - | - | - |  | - | - |
| IL-17 | - | - | - |  | - | - |
| G-CSF | - | - | - |  | - | - |
| GM-CSF | - | - | - |  | - | - |
| IFN-γ | - | - | - |  | - | - |
| MCP-1 | - | - | < 0.05 |  | - | < 0.05 |
| MIP-1β | - | - | - |  | - | - |
| TNF | - | - | - |  | < 0.05 | - |

^1^PBMC sampled from patients one month postinfection.

^2^PBMC sampled from patients 12 months postinfection.

^3^No significant difference.

^4^*P-*value

Table S2


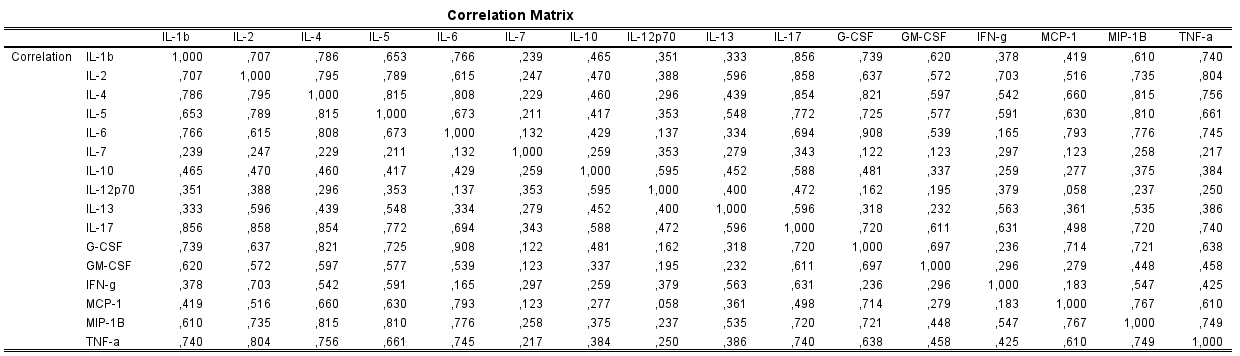


Table S3. Factor loadings of cytokines on the respective factors.

|  | Factor^1^ | | | |
| --- | --- | --- | --- | --- |
|  | 1 | 2 | 3 | 4 |
| G-CSF | 0.950 | 0.085 | 0.128 | 0.003 |
| IL-6 | 0.945 | 0.047 | 0.106 | 0.027 |
| IL-4 | 0.818 | 0.392 | 0.161 | 0.069 |
| IL-1b | 0.777 | 0.203 | 0.283 | 0.094 |
| MCP-1 | 0.742 | 0.143 | -0.021 | 0.042 |
| MIP-1B | 0.725 | 0.468 | 0.070 | 0.111 |
| TNF-a | 0.724 | 0.315 | 0.135 | 0.085 |
| GM-CSF | 0.698 | 0.143 | 0.147 | 0.012 |
| IL-17 | 0.694 | 0.504 | 0.337 | 0.146 |
| IL-5 | 0.692 | 0.505 | 0.197 | 0.034 |
| IL-2 | 0.625 | 0.616 | 0.218 | 0.056 |
| IFN-g | 0.138 | 0.922 | 0.104 | 0.120 |
| IL-13 | 0.235 | 0.729 | 0.278 | 0.087 |
| IL-12p70 | 0.019 | 0.250 | 0.941 | 0.146 |
| IL-10 | 0.390 | 0.154 | 0.662 | 0.082 |
| IL-7 | 0.085 | 0.166 | 0.180 | 0.966 |

^1^Extraction Method: Principal Component Analysis. Rotation Method: Varimax with Kaiser Normalization.

Table S4. Statistical comparisons of frequencies of cytokine-expressing CD4 memory populations between patients and vaccinees.

|  |  | CD4/TTM | | | |  | CD4/TEM | | | |
| --- | --- | --- | --- | --- | --- | --- | --- | --- | --- | --- |
| S1^1^ | S 2 | IFN-g | MIP-1B | TNF | IL-2 |  | IFN-g | MIP-1B | TNF | IL-2 |
| 1pat^2^ | Naïve | < 0.001^3^ | < 0.001 | < 0.001 | < 0.01 |  | < 0.001 | < 0.001 | < 0.01 | - |
|  | 1vc^4^ | < 0.001 | < 0.001 | < 0.01 | < 0.01 |  | < 0.001 | < 0.001 | < 0.01 | - |
|  | 2vc | - | < 0.01 | - | - |  | - | < 0.001 | - | - |
|  | 4vc | - | - | - | - |  | - | < 0.05 | - | - |
|  | 12vc | - | - | - | - |  | - | < 0.001 | - | - |
|  | 12pat | - | - | - | - |  | - | - | - | < 0.05 |
|  | HR | - | - | - | - |  | - | - | - | - |
|  |  |  |  |  |  |  |  |  |  |  |
| 12pat | Naïve | < 0.001 | < 0.001 | < 0.001 | < 0.001 |  | < 0.001 |  | - | - |
|  | 1vc | < 0.001 | < 0.001 | < 0.001 | < 0.001 |  | < 0.001 |  | - | - |
|  | 2vc | < 0.001 | < 0.001 | < 0.05 | < 0.01 |  | < 0.01 |  | - | - |
|  | 4vc | - | < 0.001 | - | - |  | - |  | - | - |
|  | 12vc | - | < 0.001 | - | - |  | < 0.01 |  | - | - |
|  | 1pat | - | - | - | - |  | - |  | - | < 0.05 |
|  | HR | - | - | - | - |  | - |  | - |  |

^1^Each row tests the null hypothesis that the Sample 1 (S1) and Sample 2 (S2) distributions are the same

^2^PBMC sampled from patients one month postinfection

^3^*P*-value

^4^PBMC sampled one week post-vaccination
